# Identification of alsterpaullone as a novel small molecule inhibitor to target group 3 medulloblastoma

**DOI:** 10.18632/oncotarget.4304

**Published:** 2015-05-28

**Authors:** Claudia C. Faria, Sameer Agnihotri, Stephen C. Mack, Brian J. Golbourn, Roberto J. Diaz, Samantha Olsen, Melissa Bryant, Matthew Bebenek, Xin Wang, Kelsey C. Bertrand, Michelle Kushida, Renee Head, Ian Clark, Peter Dirks, Christian A. Smith, Michael D. Taylor, James T. Rutka

**Affiliations:** ^1^ Division of Neurosurgery, Department of Surgery, The Hospital for Sick Children, Toronto, Canada; ^2^ Program in Cell Biology, Arthur and Sonia Labatt Brain Tumour Research Centre, The Hospital for Sick Children, Toronto, Canada; ^3^ Department of Neurosurgery, Hospital de Santa Maria, Centro Hospitalar Lisboa Norte, EPE, Lisbon, Portugal; ^4^ Instituto de Medicina Molecular, Faculdade de Medicina, Universidade de Lisboa, Lisbon, Portugal; ^5^ Program in Developmental and Stem Cell Biology, Arthur and Sonia Labatt Brain Tumour Research Centre, The Hospital for Sick Children, Toronto, Canada

**Keywords:** group 3 medulloblastoma, alsterpaullone, piperlongumine, connectivity map

## Abstract

Advances in the molecular biology of medulloblastoma revealed four genetically and clinically distinct subgroups. Group 3 medulloblastomas are characterized by frequent amplifications of the oncogene *MYC*, a high incidence of metastasis, and poor prognosis despite aggressive therapy. We investigated several potential small molecule inhibitors to target Group 3 medulloblastomas based on gene expression data using an *in silico* drug screen. The Connectivity Map (C-MAP) analysis identified piperlongumine as the top candidate drug for non-WNT medulloblastomas and the cyclin-dependent kinase (CDK) inhibitor alsterpaullone as the compound predicted to have specific antitumor activity against Group 3 medulloblastomas. To validate our findings we used these inhibitors against established Group 3 medulloblastoma cell lines. The C-MAP predicted drugs reduced cell proliferation *in vitro* and increased survival in Group 3 medulloblastoma xenografts. Alsterpaullone had the highest efficacy in Group 3 medulloblastoma cells. Genomic profiling of Group 3 medulloblastoma cells treated with alsterpaullone confirmed inhibition of cell cycle-related genes, and down-regulation of *MYC*. Our results demonstrate the preclinical efficacy of using a targeted therapy approach for Group 3 medulloblastomas. Specifically, we provide rationale for advancing alsterpaullone as a targeted therapy in Group 3 medulloblastoma.

## INTRODUCTION

Medulloblastoma is the most common malignant pediatric brain tumor consisting of at least four distinct molecular subgroups: Wingless (WNT), sonic hedgehog (SHH), Group 3 and Group 4 [1, 2]. These subgroups are characterized by divergent genetic aberrations, cytogenetic features, and distinct phenotypes including patient demographics and clinical outcome. Tumors with WNT pathway activation have the most favorable prognosis whereas Group 3 medulloblastomas have the worst outcome [3, 4]. Group 3 tumors are restricted to pediatric patients, characterized by amplification of *MYC*, and are frequently metastatic at the time of diagnosis. These tumors are particularly resistant to conventional therapies with radiation and chemotherapy, even at maximally tolerated doses, highlighting the need for novel and more effective therapeutic options [5, 6].

We used the Connectivity Map (C-MAP) database, a bioinformatic tool based on gene expression, to discover small molecule inhibitors with high likelihood of efficacy against Group 3 medulloblastomas. The C-MAP contains gene expression signatures of various cultured cancer cell lines treated with a library of small molecule compounds already approved by the Food and Drug Administration (FDA) [7, 8]. This platform links drugs, genes and diseases by measuring similarity or dissimilarity in gene expression. Using a pattern-matching algorithm, the program is able to identify drugs predicted to revert the oncogenic gene signature of a given cancer to a nonmalignant or drug-sensitive gene expression profile. Previous studies have successfully used this approach to identify compounds with the ability to modulate various biological pathways or diseases [9–13].

In this study we identified subgroup-specific signatures with genes differentially expressed between each medulloblastoma subgroup and normal cerebellum. We then selected the most frequently up- and down-regulated genes to query the C-MAP database and obtain a list of compounds likely to reverse the direction of gene expression in medulloblastoma. By limiting the C-MAP analysis to patient samples from each subgroup, we anticipate identification of *bona fide* inhibitors of each tumor subtype that might be missed if all medulloblastoma subgroup samples were merged in the analysis. Given that Group 3 medulloblastoma has the worst prognosis and current therapy results in high morbidity, identification of subgroup-specific effective compounds is a priority. Piperlongumine (PL), a natural product isolated from the fruit of the *Piper longum* and previously known to have cytotoxic properties in cancer [14], was the top candidate for non-WNT tumors. Alsterpaullone (ALP), a cyclin-dependent kinase (CDK) inhibitor, was identified as a potential therapeutic agent for Group 3 medulloblastomas.

In subsequent validation experiments we sought to validate the predictions of this *in silico* drug screen. Here we show for the first time that ALP is highly effective and selective in treating Group 3 medulloblastoma cell lines and xenografts. Furthermore, ALP reverses the Group 3 medulloblastoma gene signature and downregulates many cell cycle-related genes, including *MYC*.

## RESULTS

### The Connectivity Map identifies novel candidate drugs for medulloblastoma

To identify novel drugs with potential antitumor effect in medulloblastoma we queried the C-MAP database using the previously published gene expression signatures of the four molecular subgroups of medulloblastoma [1]. The top 20 drugs that were able to reverse the gene expression profile of each medulloblastoma subgroup are listed in Table 1. PL, a natural product derived from the plant species *Piper longum*, was the compound with the highest negative enrichment score for non-WNT medulloblastomas. The compounds identified as potential novel therapies for WNT medulloblastomas are distinct from the ones identified for non-WNT tumors. On the other hand, the drugs listed for Group 3 and Group 4 tumors are very similar.

**Table 1:** Top 20 drugs with predicted efficacy by the Connectivity Map analysis (*p*<0.05), for each medulloblastoma subgroup

WNT		
Rank	C-MAP Name	Enrichment
9	monobenzone	−0.855
24	chrysin	−0.841
5	hexamethoniumbromide	−0.806
13	simvastatin	−0.805
14	pimozide	−0.805
8	astemizole	−0.796
10	antimycin A	−0.787
43	reserpine	−0.786
46	etacrynic acid	−0.783
19	methylprednisolone	−0.774
20	chlorphenesin	−0.772
11	pyrimethamine	−0.763
12	halcinonide	−0.746
25	aminophylline	−0.745
61	esculetin	−0.745
26	3-nitropropionicacid	−0.743
29	etoposide	−0.724
3	methotrexate	−0.723
30	semustine	−0.722
31	parthenolide	−0.720
33	lomustine	−0.708

SHH		
Rank	C-MAP Name	Enrichment
66	piperlongumine	−0.919
20	trazodone	−0.916
4	luteolin	−0.915
5	phenoxybenzamine	−0.909
8	apigenin	−0.899
10	Prestwick-1084	−0.897
117	DL-thiorphan	−0.869
18	bepridil	−0.856
54	chrysin	−0.834
56	ronidazole	−0.833
23	thiostrepton	−0.831
25	puromycin	−0.830
29	repaglinide	−0.809
79	0297417-0002B	−0.803
31	GW-8510	−0.797
33	monobenzone	−0.795
87	etacrynic acid	−0.789
39	thioguanosine	−0.780
40	cyproterone	−0.780
43	Sulconazole	−0.766

Group 3		
Rank	C-MAP Name	Enrichment
3	phenoxybenzamine	−0.957
28	piperlongumine	−0.950
93	1,4-chrysenequinone	−0.892
101	DL-thiorphan	−0.885
11	monobenzone	−0.841
13	carbachol	−0.833
47	doxorubicin	−0.833
14	puromycin	−0.828
51	etacrynic acid	−0.828
16	imipenem	−0.815
63	chrysin	−0.807
18	luteolin	−0.801
19	trifluridine	−0.797
21	GW-8510	−0.796
72	0297417-0002B	−0.793
2	resveratrol	−0.785
7	levonorgestrel	−0.779
90	alsterpaullone	−0.777
30	metyrapone	−0.771
9	medrysone	−0.770

Group 4		
Rank	C-MAP Name	Enrichment
12	piperlongumine	−0.971
1	phenoxybenzamine	−0.930
13	etacrynic acid	−0.902
92	1,4-chrysenequinone	−0.896
28	chrysin	−0.865
51	0297417-0002B	−0.825
14	apigenin	−0.815
63	Prestwick-559	−0.811
16	tyloxapol	−0.810
64	doxyrubicin	−0.809
17	imipenem	−0.808
70	ronidazole	−0.803
21	Prestwick-1084	−0.801
34	sulconazole	−0.771
6	levonorgestrel	−0.759
111	milrinone	−0.759
38	protriptyline	−0.754
41	GW-8510	−0.750
43	metyrapone	−0.747
44	sulfametoxydiazine	−0.746

We then asked which compounds were specific for Group 3 medulloblastomas. On top of the list, a CDK inhibitor (ALP), a protein kinase C (PKC) inhibitor (rottlerin) and two calcium channel inhibitors (denatonium benzoate and flunarizine) showed significant negative enrichment (Table 2).

**Table 2:** Top 15 drugs specific for Group 3 medulloblastomas, as predicted by the Connectivity Map analysis (*p* < 0.05)

Rank	C-MAP name	Enrichment	Drug category
96	alsterpaullone	−0.765	CDK inhibitor
130	rottlerin	−0.725	PKC inhibitor
69	denatonium benzoate	−0.701	calcium channel inhibitor
104	flunarizine	−0.664	calcium channel inhibitor
107	bupropion	−0.661	dopamine receptor antagonist
117	pyridoxine	−0.651	pyridoxal kinase agonist
31	flunisolide	−0.644	phospholipase A2 inhibitor
125	etamsylate	−0.643	prostaglandin inhibitor
135	prenylamine	−0.635	calcium channel inhibitor
139	practolol	−0.631	beta-adrenergic antagonist
140	betaxolol	−0.629	beta-adrenergic antagonist
144	propylthiouracil	−0.626	thyroid peroxidase inhibitor
136	lorglumide	−0.57	colecystokinin antagonist
138	amiodarone	−0.569	calcium channel inhibitor
113	PNU-0251126	−0.549	not assessed

NOTE: The compounds were ranked based on negative enrichment score.

CDK, cyclin-dependent kinase; PKC, protein kinase C

### C-MAP candidate drugs piperlongumine, alsterpaullone, rottlerin and flunarizine reduce proliferation of Group 3 medulloblastoma cell lines

To validate the results of our C-MAP analysis we selected PL (the best candidate for non-WNT medulloblastomas) and the top three drugs predicted to be specific for Group 3 medulloblastomas (alsterpaullone, rottlerin and flunarizine).

**Figure 1:**
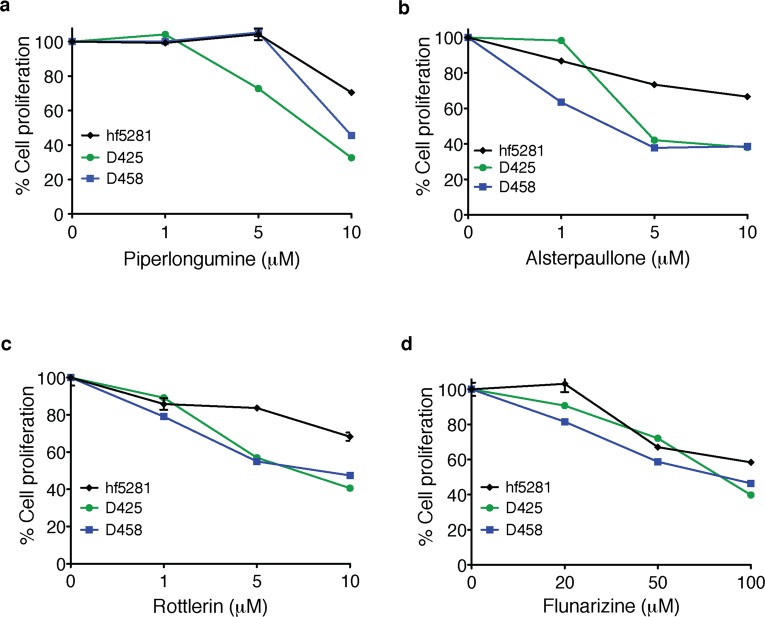
Cytotoxic effect of piperlongumine, alsterpaullone, rottlerin and flunarizine in Group 3 medulloblastoma cell lines Established medulloblastoma cell lines (D425 and D458) and a fetal neural stem cell line (hf5281) were treated with various concentrations of **a.** piperlongumine, **b.** alsterpaullone, **c.** rottlerin and **d.** flunarizine for 48 hours. Cell viability was measured by MTS assay. Data represent mean of triplicates ± SEM.

We examined the effects of each drug on the proliferation of two established Group 3 medulloblastoma cell lines, D425 and D458, and a fetal normal human brain culture (hf5281) [15–19]. PL and rottlerin (RTL) treatment for 48 hours reduced cell proliferation in medulloblastoma cells at 5 μM (Figure 1a and 1c) whereas ALP treatment showed the same efficacy at 1 μM (Figure 1b). Treatment with flunarizine (FZ) decreased cell proliferation at higher concentrations (50 and 100 μM) (Figure 1d). When normal human brain cells (hf5281) were incubated with PL, ALP and RTL there was little reduction in cell proliferation, even at the highest concentration tested of 10 μM, thus indicating that these compounds may have selective killing properties to medulloblastoma tumor cells.

### *In vivo* antitumor effect of piperlongumine, alsterpaullone and rottlerin in Group 3 medulloblastomas

**Figure 2:**
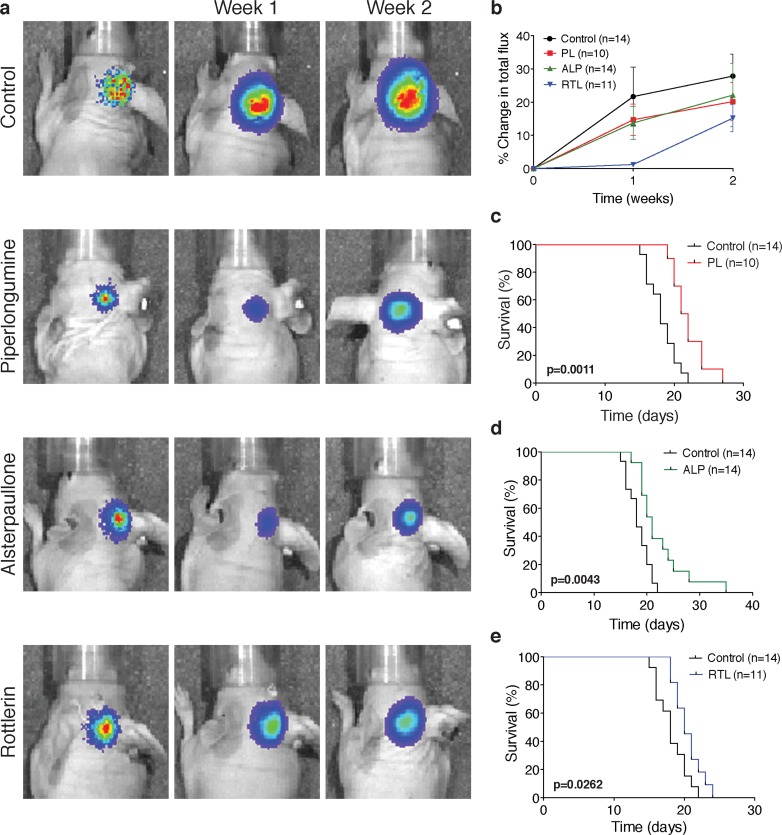
Piperlongumine (PL), alsterpaullone (ALP) and rottlerin (RTL) reduce tumor growth and increase survival in medulloblastoma xenografts **a.** Representative bioluminescence imaging of D458 cerebellar xenografts treated with vehicle control (10% DMSO, *n* = 14), PL (50 mg/kg, s.c., daily for 2 weeks; *n* = 10), ALP (30 mg/kg, s.c., daily for 2 weeks; *n* = 14) and RTL (20 mg/kg, s.c., every other day for 2 weeks; *n* = 11). **b.** Connectivity Map predicted drugs reduce medulloblastoma growth as denoted by a smaller change in total photon flux. Data represent group means ± SEM. **c.**–**e.** Kaplan-Meier survival curves demonstrate that mice harboring orthotopic D458 medulloblastoma xenografts have an increased survival after treatment with PL (*p* = 0.0011), ALP (*p* = 0.0043) and RTL (*p* = 0.0262). Survival differences were calculated using a log-rank test.

We next investigated the efficacy of PL, ALP, RTL and FZ in established medulloblastoma xenografts representative of Group 3 medulloblastomas. D458 cells expressing luciferase were implanted in the right cerebellum of nude mice and bioluminescence imaging was performed at 6 days post inoculation. Animals with a detectable signal were treated by subcutaneous injection with PL (50 mg/kg, daily for 2 weeks), ALP (30 mg/kg, daily for 2 weeks), RTL (20 mg/kg, every other day for 2 weeks), FZ (50 mg/Kg, daily for 2 weeks) or vehicle control (10% DMSO). Marked reduction in medulloblastoma growth was observed in mice treated with PL, ALP and RTL when compared to DMSO-treated controls, as confirmed by bioluminescence imaging (Figure 2a and 2b) and by histological examination (H&E stain) of the brains (Supplementary Figure 1a). A significant increase in survival was also seen in mice treated with PL (Figure 2c; *p* = 0.0011), ALP (Figure 2d; *p* = 0.0043) and RTL (Figure 2e; *p* = 0.0262). As expected by the *in vitro* effects of FZ in cell proliferation, only seen at very high concentrations, this drug was not able to prolong survival of mice bearing medulloblastoma xenografts (Supplementary Figure 1b).

**Figure 3:**
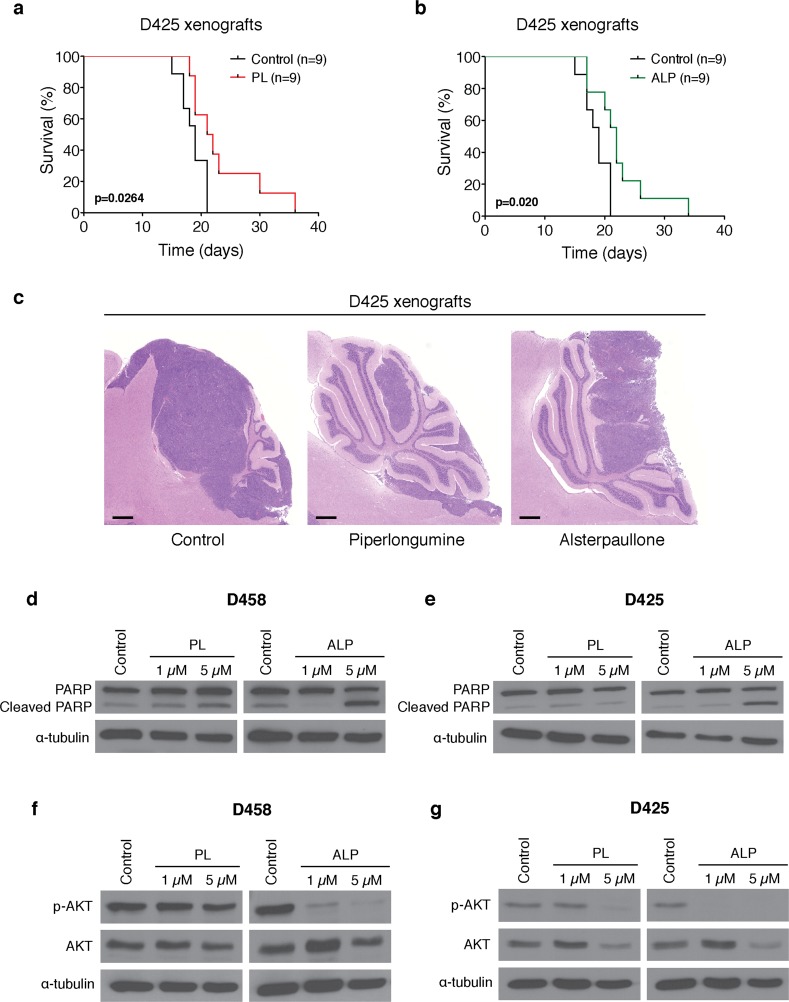
Piperlongumine and alsterpaullone increase survival of D425 medulloblastoma xenografts Kaplan-Meier estimate displays survival of nude mice with D425 cerebellar xenografts treated with **a.** piperlongumine (PL) or **b.** alsterpaullone (ALP). Survival differences were calculated using a log-rank test. **c.** Representative H&E staining demonstrates reduction in medulloblastoma growth after treatment with PL and ALP, when compared to the control group. Scale bar: 500 μm. Piperlongumine and alsterpaullone induce apoptosis and inhibit AKT pathway activation. Representative Western blots demonstrating **d.** and **e.** induction of apoptosis and **f.** and **g.** AKT pathway inhibition after piperlongumine (PL) and alsterpaullone (ALP) treatment for 48h in D458 and D425 medulloblastoma cells.

We then tested the two most promising drugs, PL and ALP, in nude mice with D425 cerebellar xenografts and showed that both drugs significantly increase survival (Figure 3a and 3b; *p* < 0.05) and reduce medulloblastoma growth (Figure 3c). Collectively, these results confirm that the C-MAP top predicted drugs for Group 3 medulloblastomas are effective in treating orthotopic mouse models of the disease.

To determine the mechanisms by which PL and ALP exert their antitumor effect, we treated D425 and D458 medulloblastoma cells with both drugs for 48h and assessed apoptosis and AKT pathway inhibition. Western blot analysis showed that ALP induced apoptosis (Figures 3d and 3e) and potently inhibited AKT pathway activation at lower concentrations than PL (Figures 3f and 3g).

### Alsterpaullone inhibits MYC and other cell cycle related genes

**Figure 4:**
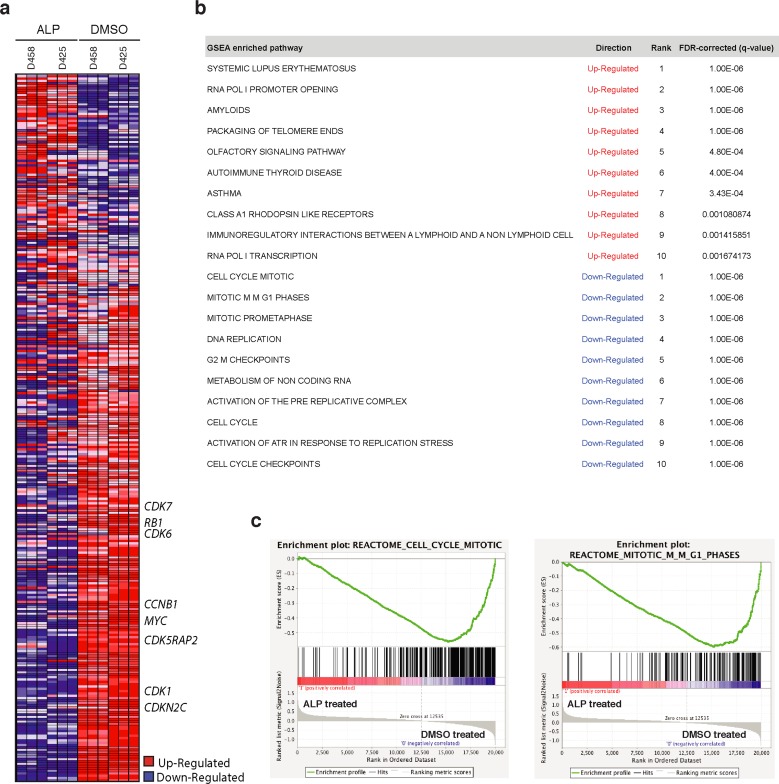
mRNA profiling of Group 3 medulloblastoma cell lines treated with alsterpaullone demonstrates down-regulation of cell cycle related genes, including the *MYC* oncogene **a.** Heatmap illustrating the genes up and down-regulated following treatment with alsterpaullone (ALP) in two Group 3 medulloblastoma cell lines. *MYC* and other genes involved in cell cycle are down-regulated by ALP. **b.** Table of the top 10 gene sets up-regulated or down-regulated by ALP. **c.** Enrichment plots showing down-regulation of cell cycle related gene sets.

To identify downstream transcriptional events induced by ALP, we performed genomic profiling of Group 3 medulloblastoma cell lines (D425 and D458), after treatment for 48 hours. When compared to DMSO treated medulloblastoma cells, ALP treated cells showed down-regulation of genes involved in cell cycle, including *MYC* (Figure 4a). Gene Set Enrichment Analysis (GSEA) was performed with gene sets compiled from the National Cancer Institute (NCI), Gene Ontology (GO), Kyoto Encyclopedia of Genes and Genomes (KEGG), Protein Families (PFAM) and Biocarta pathway databases. To visualize significant gene sets (FDR < 0.05; *p* < 0.01) as interaction networks, Cytoscape and Enrichment Map were used. The top-scoring gene sets down-regulated by ALP were mainly cell cycle-related transcriptional signatures (Figure 4b and 4c). In addition, ALP also inhibits several cancer-related networks (namely MTOR signaling, PTEN signaling, RAS signaling, Aurora kinase signaling, insulin signaling) and other biological processes including RNA processing, transport and splicing, DNA repair, chromatin organization and histone modifications, carbon metabolism and phosphatase activity (Supplementary Figure 2). Gene sets up-regulated by ALP involve the inflammatory response (TNF signaling, MHC antigen presentation) and tissue regeneration (wound response and coagulation cascade, tissue morphogenesis, muscle contraction), as well as olfactory signaling, STAT signaling, alkene metabolism, lipid transport and neuropeptide receptor activity (Supplementary Figure 2).

Interestingly, when we queried the C-MAP database using the gene expression profiling of D425 and D458 medulloblastoma cells after treatment with ALP and PL, we found that only ALP was able to significantly reverse gene expression as determined by the high positive enrichment score (0.995) and low *p*-value (*p* < 0.000001) (Supplementary Figure 3 and Supplementary Figure 4).

## DISCUSSION

We have used an *in silico* gene expression-based screening for drug discovery in medulloblastoma. Using the Connectivity Map (C-MAP) algorithm, we identified the CDK inhibitor ALP as a compound with the ability to reverse the gene expression signature of Group 3 medulloblastomas and, therefore, with potential specific antitumor effect. We validated the results obtained with C-MAP by demonstrating the efficacy of ALP both in *in vitro* and *in vivo* models of Group 3 medulloblastoma. Our study provides evidence for the use of chemical genomics to identify modulators of biological processes that drive the medulloblastoma subgroups.

The C-MAP analysis, using the gene expression signatures of the four molecular subgroups of medulloblastoma, led to interesting observations. The drugs predicted for the WNT subgroup were distinct from the other subgroups. Strikingly, methylprednisolone (a steroid known to have a beneficial effect in brain tumors) and four chemotherapeutic agents currently in use for medulloblastoma treatment (etoposide, methotrexate, semustine and lomustine) were only listed in the WNT subgroup, which has the best response to treatment and the best outcome. On the other hand, PL was the top ranked compound for non-WNT medulloblastomas suggesting the ability of this compound to reverse common biological processes in these tumors. The predictions for Group 3 and Group 4 medulloblastomas included several shared compounds, in agreement with the genetic similarity between the two subgroups. Doxorubicin, a well-known chemotherapeutic agent but not used to treat medulloblastoma, was identified as a drug with potential efficacy for both Group 3 and Group 4 tumors.

The top ranked compound with predicted specificity for Group 3 medulloblastoma was ALP, a potent inhibitor of CDK1/cyclin B and CDK 5. The cell-cycle kinases play an important role in normal brain development and their deregulation is associated with aberrant division and uncontrolled proliferation in various cancers, including malignant brain tumours [20, 21]. In cerebellar development, cyclins D1 and D2 play a crucial role in the postnatal expansion of the granule neuron precursors (GNP) in the external granule layer (EGL). As these cells exit the cell cycle and begin to differentiate, they migrate inwards through the Purkinje cell layer to form the internal granule layer (IGL) of the adult cerebellum [22, 23]. The SHH pathway regulates the GNPs proliferation inducing the expression of the transcription factor GLI1, which up-regulates the expression of cyclin D1 and cyclin D2, via N-MYC [24, 25]. Removal of both cyclins D1 and D2 in animal models results in severe cerebellar hypoplasia due to decreased proliferation and increased apoptosis of GNPs [26]. In *Ptch^+/−^* mice, known to develop spontaneous cerebellar medulloblastomas, loss of cyclin D1 (*Ptch^+/−^; Ccnd1^−/−^*) significantly reduces the incidence of tumors [27]. On the other hand, loss of CDK inhibitors, *Ink4c* or *Ink4d*, triggers medulloblastoma formation in p53-null mice [28]. A recent study has shown that CDK5 is also required for the normal development of the cerebellum. Cdk5 conditional knockout mice display a smaller cerebellum and a profound disturbance in migration of granule cells [29].

Members of the Cyclin/CDK complex are frequently amplified or up-regulated in human medulloblastoma. Li *et al.* described amplifications of the *CDK4*, *CDK6*, *CCND1* (or cyclin D1) and *CCND2* (or cyclin D2) genes in primary medulloblastomas [30], raising the possibility of using CDK inhibition to suppress medulloblastoma formation. Interestingly, it has been shown in mouse models of lymphoma and hepatoblastoma that MYC sensitizes tumor cells to undergo apoptosis in response to CDK1 inhibition, through a mechanism independent of *p53* status [31]. Due to the limited success in developing small molecule inhibitors of MYC, targeting important cellular processes, such as the cell cycle, may prove to be a viable therapeutic strategy in MYC-dependent tumors, including Group 3 medulloblastomas.

Previous studies have shown the antitumor effects of ALP through induction of apoptosis in breast cancer and leukemia cells [32–34]. We report for the first time the cytotoxic effects of ALP in medulloblastoma. ALP effectively decreased cell proliferation and induced apoptosis through AKT pathway blockade. In mouse xenografts of Group 3 medulloblastoma, treatment with ALP significantly reduced tumor growth and improved survival. Given that Paullones, including alsterpaullone, have been identified as CDK1/CDK2/CDK5 and GSK3B inhibitors, we examined the gene expression profiles of the CDK genes in the Group 3 medulloblastoma cell lines. We found that multiple CDK genes are upregulated in untreated control medulloblastoma cells, which were significantly inhibited in cells treated with alsterpaullone. This supports the notion that the mechanism of action of alsterpaullone is the targeting of CDKs which results in the growth inhibition and apoptosis that we observed. Future studies include characterization of CDK regulation in Group 3 medulloblastoma.

Taken together, these experimental results confirm the value of the C-MAP algorithm by establishing that ALP may serve as a Group 3-specific drug. In fact, we demonstrate that ALP down-regulates several cell cycle related genes, including *MYC*, and, unlike PL, treatment with ALP reverses the gene expression profile of Group 3 medulloblastoma cell lines. Our chemical genomics study provides a rationale for advancing alsterpaullone as a targeted therapy in Group 3 medulloblastoma.

## MATERIALS AND METHODS

### Connectivity map analysis

The subgroup-specific gene expression profiles of primary medulloblastomas were obtained from previously published datasets [1]. A list of genes differentially expressed between each medulloblastoma subgroup and normal cerebellum was obtained and the top 200 up- and downregulated genes were selected to query the C-MAP database. Compounds with a negative enrichment score, which implies the ability to reverse the direction of expression of the gene signature of interest, and a p-value inferior to 0.05 were recorded as potential therapeutic agents for medulloblastoma [7, 8].

### Medulloblastoma cell lines

The medulloblastoma cell lines (D425 and D458) were kindly provided by Dr. Annie Huang, Hospital for Sick Children, Toronto, Canada. D458-GFP/Luciferase cells were generated as described previously [17].

### Cell proliferation assays

D425 and D458 cells were grown as suspension cultures and seeded in 96-well microplates at 10,000 cells per well. hf5281 was grown as an adherent culture and seeded in 96-well microplates at 4,000 and 5,000 cells per well. Cells were treated for 48h with different concentrations of piperlongumine (PL; INDOCINE Chemical Company), alsterpaullone (ALP; A.G. Scientific), rottlerin (RTL; A. G. Scientific) and flunarizine (FZ; Sigma) or DMSO (control). Cell viability was determined by MTS (3-(4,5-dimethylthiazol-2-yl)-5-(3-carboxymethoxyphenyl)-2-(4-sulfophenyl)-2H-tetrazolium) and the absorbance measured at 490 nm (CellTiter 96 Aqueous One Solution Reagent; Promega). Three independent experiments were performed with 16 repeats per treatment condition.

### Medulloblastoma xenografts

All mouse studies were approved by the Institutional Animal Care and Use Committee of the University of Toronto and the Hospital for Sick Children, in Toronto, and performed in accordance to their policies and regulations.

Medulloblastoma intracranial xenografts were established in 5-6 week old athymic nude mice (Charles River Laboratories). Medulloblastoma cells (250,000 D425 and D458 cells) were implanted in the right cerebellum of mice. Six days after cell inoculation, animals were randomized into treatment cohorts, which included subcutaneous injections with vehicle control (10% DMSO), PL (50 mg/kg, daily for 2 weeks), ALP (30 mg/kg, daily for 2 weeks), RTL (20 mg/kg, every other day for 2 weeks) or FZ (50 mg/Kg, daily for 2 weeks).

In animals bearing D458-GFP/Luciferase xenografts, bioluminescence imaging was performed at 6 days after intracranial injection. Mice with a detectable signal were included in the study and tumor growth was monitored at one-week intervals using the IVIS Spectrum Optical In-vivo Imaging System (Caliper Life Sciences).

Animals with progressive neurological signs or weight loss greater than 20% were euthanized and the brains harvested and fixed in 10% formalin.

### Immunoblotting

Cell lysates were prepared by adding RIPA buffer (Sigma) containing protease inhibitors (F. Hoffman-La Roche AG), 0.2 M sodium orthovanadate, 0.2 M sodium pyrophosphate and 0.2 M sodium fluoride. The Pierce BCA Protein Assay Kit (Thermo Scientific) was used to determine protein concentration. Proteins were separated on 7.5% or 10% SDS-PAGE gels and transferred to PVDF membranes using a semidry transfer apparatus (Bio-Rad). The following antibodies were used: PARP (1:1,000; Cell Signaling), AKT (1:1,000; Cell Signaling), phospho-AKT (1:2,000; Ser473, Cell Signaling), α-tubulin (1:1,000; Cell Signaling), anti-mouse IgG conjugated to horseradish peroxidase (1:5,000; Amersham Biosciences) and anti-rabbit IgG conjugated to horseradish peroxidase (1:5,000; Cell Signaling).

### RNA extraction and gene expression analysis

D425 and D458 medulloblastoma cells were treated with 5 μM of PL, 5 μM of ALP or DMSO (control) for 48h. RNA isolation was performed using the RNeasy Mini Kit (Qiagen) and gene expression data were generated using the Human PrimeView Arrays. Gene Set Enrichment Analysis (GSEA) was performed using gene sets from the National Cancer Institute (NCI), Gene Ontology (GO), Kyoto Encyclopedia of Genes and Genomes (KEGG), Protein Families (PFAM) and Biocarta pathway databases. Significant gene sets were identified (FDR < 0.05; *p* < 0.01) and visualized in Cytoscape and Enrichment Map software.

A list of genes up- and down-regulated by ALP in D425 and D458 medulloblastoma cells was generated. The top 200 genes were selected to query the C-MAP database and to determine if alsterpaullone and piperlongumine were able to reverse the gene expression profile of Group 3 medulloblastoma cells (*p* < 0.05).

### Statistical analysis

The Kaplan-Meier estimate and a log-rank test were used to generate survival curves. Experiments were performed in triplicate and results were expressed as mean ± SEM. Statistical analysis was performed using GraphPad Prism 5 Software. A p*-*value inferior to 0.05 was considered as significant.

## SUPPLEMENTARY MATERIAL FIGURES


