# Supplementary material for: Identification of alsterpaullone as a novel small molecule inhibitor to target group 3 medulloblastoma
Source: Oncotarget. 2015 May 28;6(25):21718–29. doi: 10.18632/oncotarget.4304 (PMC4673298; doi:10.18632/oncotarget.4304)
Supplement: Supplementary file 1 [file oncotarget-06-21718-s001.pdf]

Identification of alsterpaullone as a novel small molecule inhibitor to target group 3 medulloblastoma

Supplementary Material

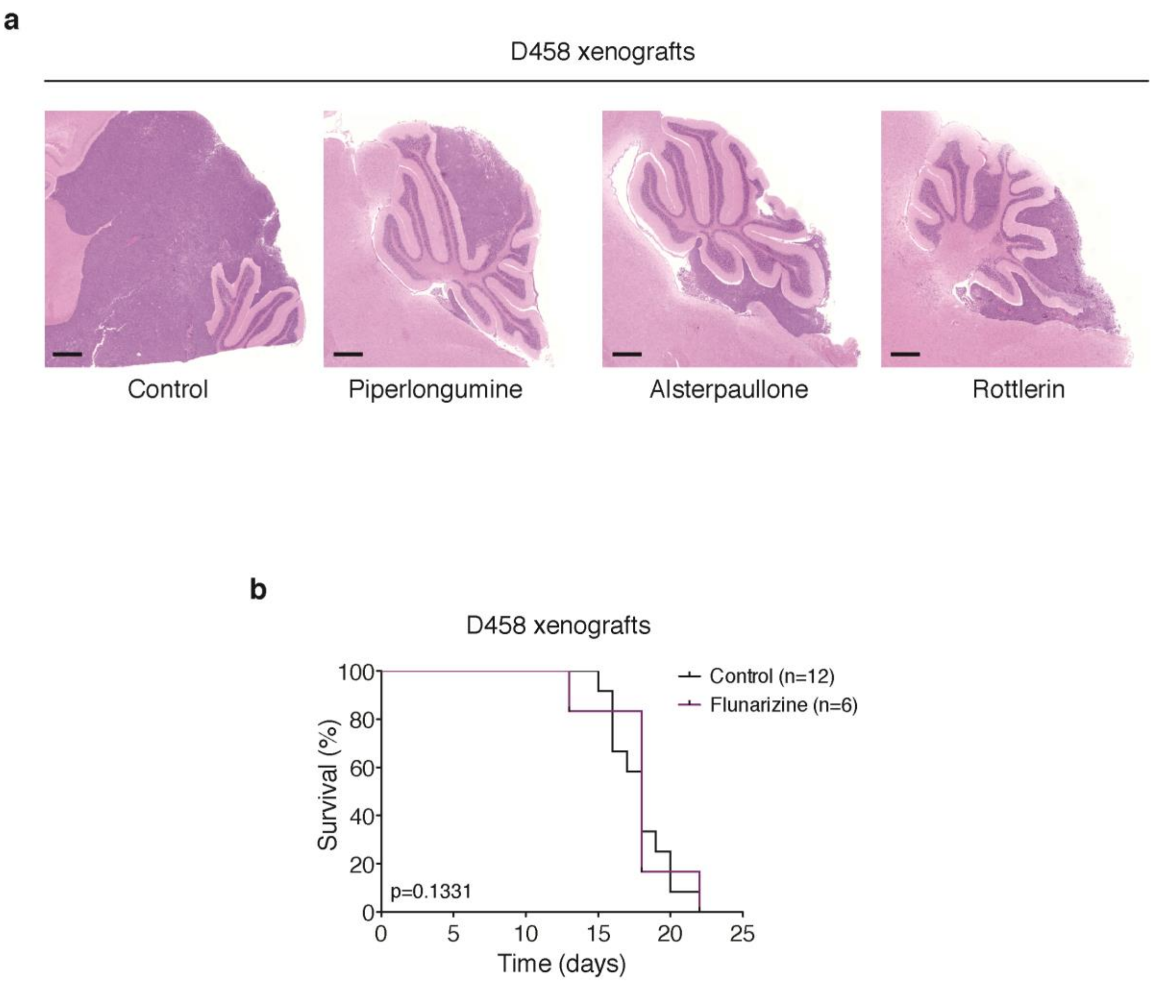

Supplementary Figure 1. **a** Representative H&E staining demonstrates that mice bearing D458 tumors treated with piperlongumine, alsterpaullone and rottlerin have smaller medulloblastomas in the cerebellum. Scale bar: 500 μm. **b** Kaplan-Meier survival curves of D458 medulloblastoma xenografts treated with flunarizine (50 mg/Kg, daily for 2 weeks; n = 6) or vehicle control (10% DMSO; n = 12). Survival differences were calculated using a log-rank test.

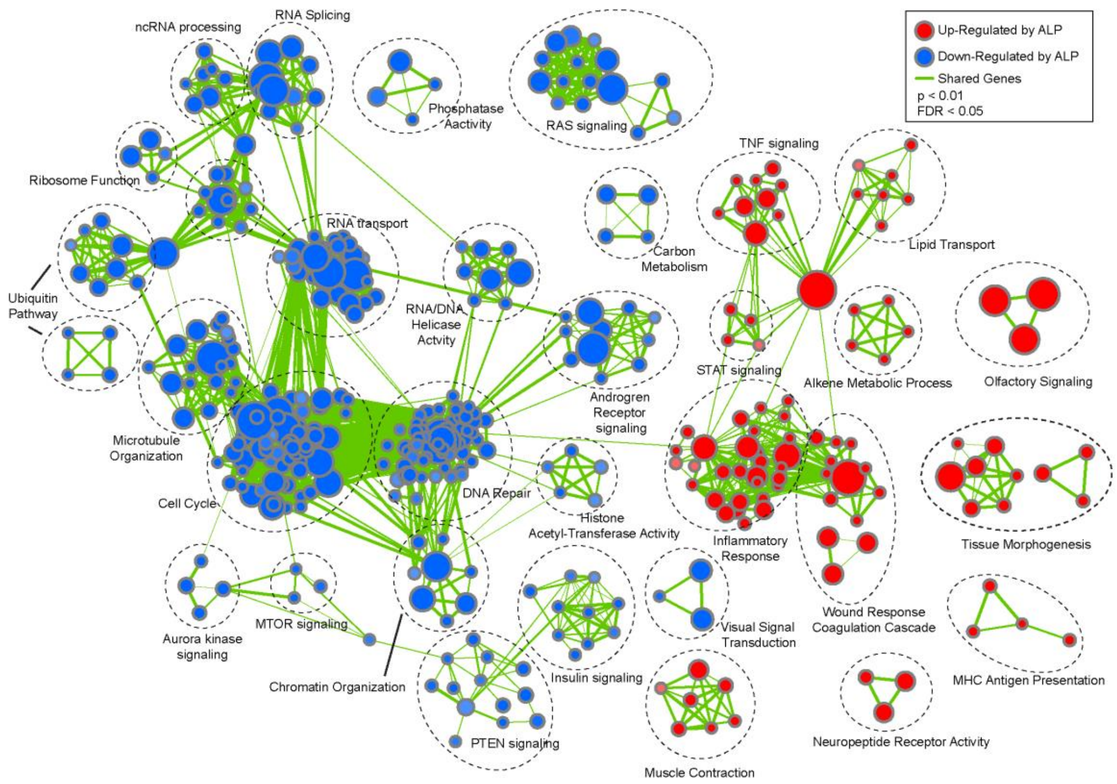

Supplementary Figure 2. Biological pathways and processes up- and down-regulated by alsterpaullone. Gene Set Enrichment Analysis (GSEA) comparing gene sets up- and down-regulated by alsterpaullone (ALP) in D458 and D425 medulloblastoma cells (FDR < 0.05; $p < 0.01$). Cytoscape and Enrichment Map were used for visualization of the GSEA results. The enriched gene sets were grouped by their similarity, represented as nodes, and mapped as a network. The size of each node determines the total number of genes within each gene set.

a

cmap name	mean	n	enrichment	p-value	specificity	% non-null
alsterpaullone	0.853	3	0.995	< 0.000001	0.0116	100

alsterpaullone

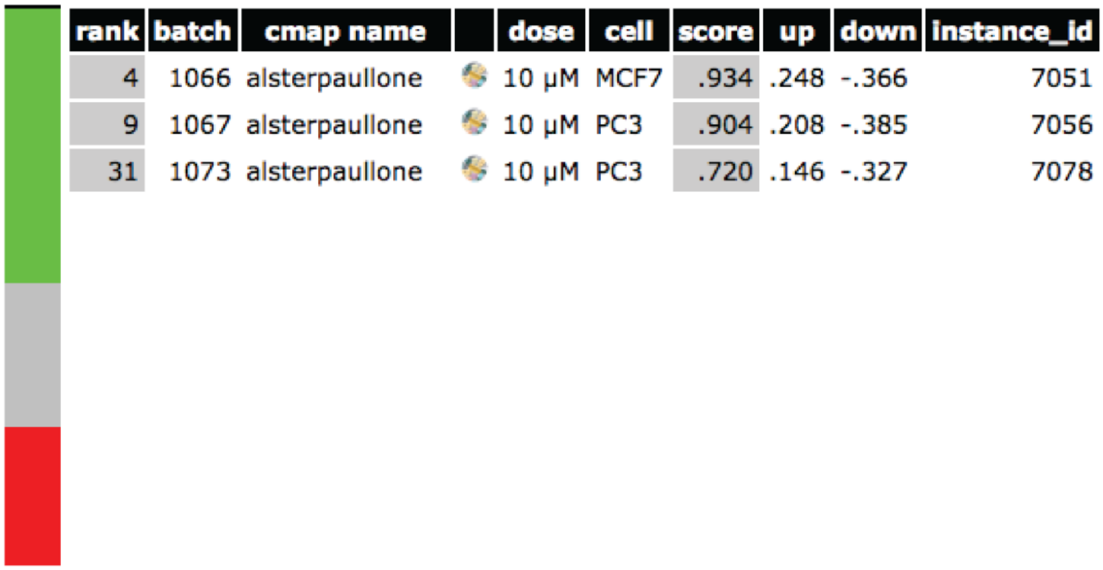

rank	batch	cmap name		dose	cell	score	up	down	instance_id
4	1066	alsterpaullone		10 µM	MCF7	.934	.248	-.366	7051
9	1067	alsterpaullone		10 µM	PC3	.904	.208	-.385	7056
31	1073	alsterpaullone		10 µM	PC3	.720	.146	-.327	7078

b

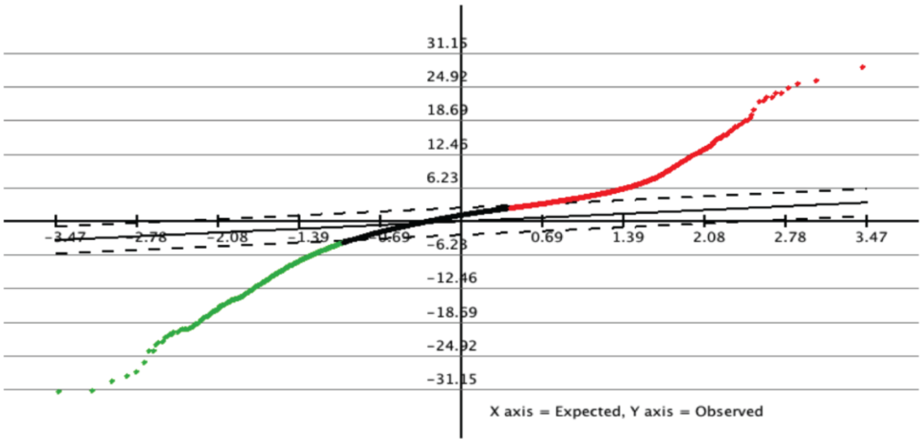

Supplementary Figure 3. Alsterpaullone reverses Group 3 medulloblastoma gene expression signature as shown by (a) the C-MAP analysis and (b) the Significance Analysis of Microarrays (SAM) plot.

a

cmap name	mean	n	enrichment	p-value	specificity	% non-null
piperlongumine	-0.061	2	-0.474	0.59627	0.7342	50

piperlongumine

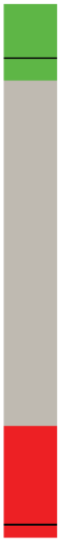

rank	batch	cmap name		dose	cell	score	up	down	instance_id
614	641	piperlongumine		13 µM	HL60	.499	.067	-.095	1764
5943	662	piperlongumine		13 µM	MCF7	-.621	-.128	.130	2757

b

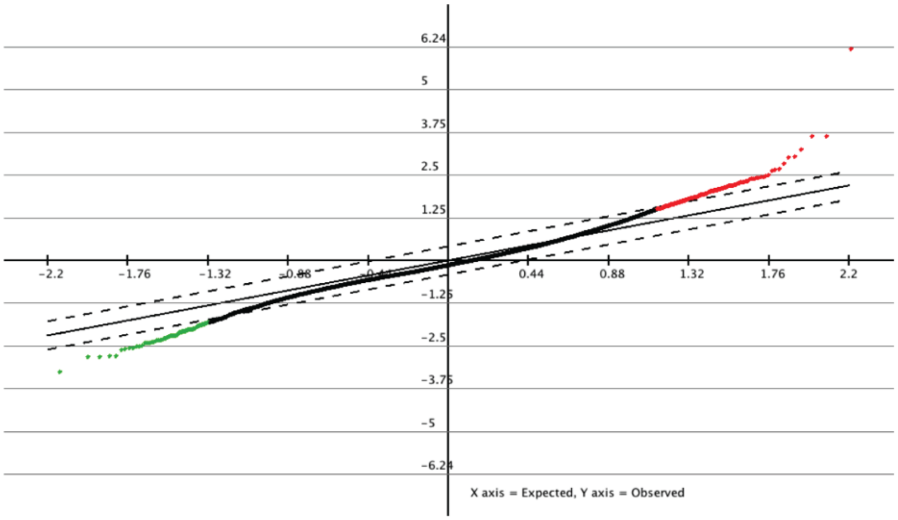

Supplementary Figure 4. The gene expression profile of Group 3 medulloblastomas is not affected by piperlongumine as determined by (a) the C-MAP analysis and (b) the Significance Analysis of Microarrays (SAM) plot.
